# Exploring the Impacts of Housing Instability on Intimate Partner Violence, Adverse Health and Risk Behaviours Among Women

**DOI:** 10.1111/jan.70694

**Published:** 2026-07-29

**Authors:** Claudia L. Michaels, Jianfang Liu, Jean Jimenez, Maeve Brin, Nancy Glass, Amy K. Johnson, Mirjam‐Colette Kempf, Rebecca Schnall

**Affiliations:** ^1^ Columbia University School of Nursing New York New York USA; ^2^ Johns Hopkins University School of Nursing Baltimore Maryland USA; ^3^ Ann & Robert H. Lurie Children's Hospital of Chicago Chicago Illinois USA; ^4^ Northwestern University Chicago Illinois USA; ^5^ University of Alabama at Birmingham Schools of Nursing, Public Health, Medicine Birmingham Alabama USA

**Keywords:** housing instability, intimate partner violence, mental health, sexually transmitted infections, substance use, women

## Abstract

**Aim:**

To examine associations between housing instability and intimate partner violence (IPV) exposure, adverse mental health, sexually transmitted infection (STI) and HIV incidence, and substance use among women across the U.S. and its territories.

**Design:**

Cross‐sectional analysis of data from the American Women: Assessing Risk Epidemiologically (AWARE) study.

**Methods:**

Survey data and STI/HIV results from 1626 women between July 2023 and March 2025 were used to conduct multivariable regression analyses adjusted for sociodemographic factors.

**Results:**

Sixteen percent of women reported current housing instability. Housing instability was associated with higher levels of IPV (B = 2.43, 95% CI = 1.68–3.18), depression (B = 3.25, 95% CI = 2.61–3.88), and anxiety (B = 3.38, 95% CI = 2.60–4.15), and increased likelihood of STI/HIV diagnosis (RRR = 1.85, 95% CI = 1.16–2.97). Unstably housed women were more than twice as likely to report hazardous use of marijuana (OR = 2.02, 95% CI = 1.38–2.95), stimulants (OR = 2.31, 95% CI = 1.45–3.69), and opioids (OR = 2.72, 95% CI = 1.33–5.56), although no association was observed for alcohol use.

**Conclusion:**

These findings align with research linking housing instability to poor health and violence victimization.

**Impact:**

These findings are relevant to nurses, advanced practice providers, public health practitioners, HIV/STI prevention programs, behavioural health clinicians, and housing‐service providers who care for women in emergency departments, community health centres, sexual health clinics, substance use treatment programs, and shelter‐based or outreach settings. Results support integrating housing instability screening with IPV, mental health, substance use, and HIV/STI prevention services, and strengthening referral pathways to trauma‐informed housing support, safety planning, behavioural health care, harm reduction services, and sexual health services.

**Patient or Public Contribution:**

Not applicable.

**Abbreviations**
ADI: area deprivation index
AUDIT‐C: alcohol use disorders identification test‐consumption
AWARE: American women assessing risk epidemiologically
CASR‐SF: revised short form composite abuse scale
CESD‐R‐10: centre for epidemiologic studies depression scale‐revised
CI: confidence interval
FIPS: federal information processing standard
GAD‐7: generalized anxiety disorder‐7
HIV: human immunodeficiency virus
IPV: intimate partner violence
IRB: institutional review board
OR: odds ratio
RRR: relative risk ratio
STI: sexually transmitted infection
TAPS: tobacco, alcohol, prescription medication, and other substance use
VIF: variance inflation factor

## 1 Introduction

Housing instability, ranging from homelessness to difficulty paying rent, overcrowding, or living in unsafe conditions, is a pressing and escalating public health concern in the United States (U.S. Department of Health and Human Services 2020). Structural factors such as gentrification, foreclosure, discrimination, residential segregation, and natural disasters further contribute to housing instability (Swope and Hernández 2019). Recent national reports show dramatic increases in housing cost burdens and homelessness; in 2024, over 771,000 people were unhoused, an 18% rise from 2023 (Sousa and Henry 2024). These trends are alarming given the well‐documented association between unstable housing and adverse health outcomes, including infectious diseases, chronic illness, mental health disorders, and violence victimization (Swope and Hernández 2019).

Cisgender women (hereafter referred to as women) face unique housing‐related vulnerabilities. In 2024, women represented 39% of the U.S. homeless population, with over a quarter living unsheltered (Sousa and Henry 2024). For many women, intimate partner violence (IPV) is both a precipitant and consequence of housing instability. Abusive partners may restrict access to financial resources, impede employment, and isolate women from social support, increasing the risk of homelessness (Hing et al. 2021). Structural inequities including the gender wage gap and racial discrimination in rental markets further compound these risks for women, particularly Black, Latina and Indigenous women (Anderson et al. 2018). Housing instability also increases opportunities for revictimization and heightens exposure to sexual risk behaviours and STIs (Jeffers et al. 2022). Prior evidence demonstrates that housing instability and IPV jointly contribute to heightened risk for depression, anxiety, STIs and substance use (Chiaramonte et al. 2022; Goodman‐Williams et al. 2023).

As homelessness continues to rise across the U.S., including more than 30% increases in at least 11 states since 2015 (Soucy et al. 2024), there is an urgent public health need to understand how housing instability intersects with women's health. Prior research has focused on population subgroups, whereas few studies have investigated the impacts of housing instability on IPV exposure, substance use, and mental health conditions among women at the national level. This study is guided by a conceptual framework integrating social determinants of health, syndemic theory, and the social ecological model (Baral et al. 2013; Singer et al. 2017). Housing instability represents a structural determinant that shapes women's exposure to violence, mental health stressors, and substance use at the individual, interpersonal, community, and policy levels (Daoud et al. 2016). These conditions interact synergistically, increasing vulnerability to the syndemic of HIV and other STIs (Grome et al. 2021). Understanding these intersecting pathways is critical for identifying intervention points to improve women's health across multiple outcomes.

## 2 Methods

### 2.1 Study Design and Procedures

Data for this study were collected as part of the American Women: Assessing Risk Epidemiologically (AWARE) study. AWARE is the largest national virtual cohort of women focused on HIV/STI vulnerability, and collects detailed sociodemographic, behavioural, social network, and HIV/STI data from women across the U.S. and its territories, including biological samples collected through home‐based specimen kits (Schnall et al. 2024). In this analysis, we examine associations between housing instability and IPV, mental health symptoms, substance use, and HIV/STI outcomes. Understanding these relationships can inform interventions aimed at disrupting the pathways linking housing instability to poor health outcomes among women.

Participants were recruited primarily through national social media advertisements on Facebook and Instagram, platforms that are widely used among women in the U.S. (Gottfried 2024), and that included a link to an online screening survey. Participants were eligible if they (1) were ≥ 14 years old; (2) spoke English and/or Spanish; (3) identified as cisgender women; (4) lived in the U.S. or its territories; (5) were HIV‐negative; (6) reported recent condomless sex with a male partner; and (7) had at least one behavioural risk factor for HIV acquisition (Schnall et al. 2024). Given that most new HIV cases in the U.S. are attributable to heterosexual contact (Centers for Disease Control and Prevention 2024), we excluded women who did not report recent heterosexual contact. Transgender women were excluded because mechanisms of HIV risk differ meaningfully from those among cisgender women (Becasen et al. 2019). Screening and enrolment data from the AWARE study are presented in Figure 1.

**FIGURE 1:**
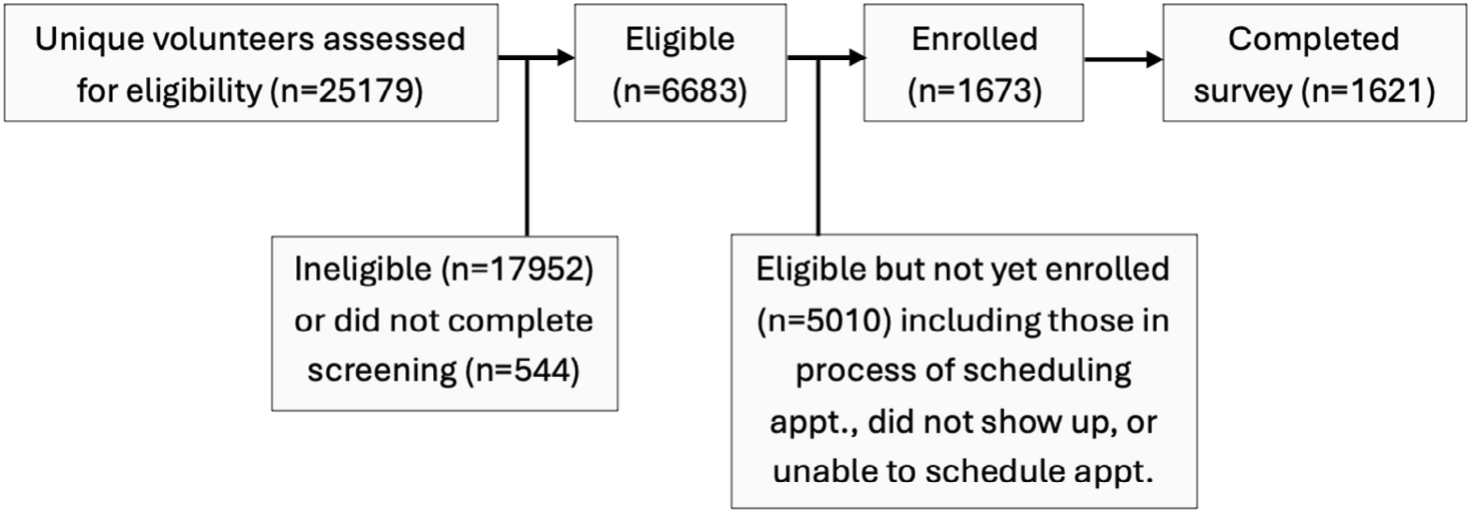
Screening and enrolment of study participants.

Participants provided addresses where they could safely receive mail for shipment of study kits and completed survey and biospecimen collection procedures via secure Zoom calls with staff. Women were observed completing study procedures in diverse environments, including homes, cars, and public or shared spaces (e.g., college dorm room, friend's home, gas station bathroom). Blood and swab samples were tested for HIV, syphilis, chlamydia, gonorrhoea, and trichomonas at the Emory University Center for AIDS Research. The present study utilises baseline data from the AWARE study.

### 2.2 Ethical Considerations

Study procedures were approved by the Columbia University IRB (IRB‐AAAU2650) on 4 August 2022, and participants provided electronic written consent for participation in all study activities.

### 2.3 Measures

Housing instability was broadly defined to include women who were currently unhoused (including living in a shelter, on the street, in a car, or staying with others) or who were currently worried about losing their housing.

Each participant was geocoded with a 12‐digit Federal Information Processing Standard (FIPS) code based on the address she provided to study staff, which was used to link survey data to the nationally ranked Area Deprivation Index (ADI) (Kind and Buckingham 2018), a validated, census‐derived composite measure of socioeconomic disadvantage at the block group level. Area deprivation is measured on a scale of 1–100, where 1 represents the least disadvantaged neighbourhoods and 100 signifies the most disadvantaged.

We measured IPV using the Revised Short Form Composite Abuse Scale (CASR‐SF) (Ford‐Gilboe et al. 2016), which measures frequency of physical, sexual, and psychological abuse and generalized partner violence in the past 12 months. Among women who reported currently being in a relationship, we calculated the count of positive IPV items by summing the ‘yes’ responses across 15 IPV items. We treated each item as a binary variable because many women experiencing an item did not indicate its frequency, resulting in a high rate of missing data in the ordinal scale of frequency. Subsequently, possible scores ranged from 0–15 with higher scores indicating greater frequency of IPV experiences.

We measured depression symptoms using the 10‐item Revised Centre for Epidemiologic Studies Depression Scale (CESD‐R‐10) (Andresen et al. 1994) in which participants indicate the frequency of depression symptoms in the past 7 days. Anxiety was measured using the Generalized Anxiety Disorder‐7 (GAD‐7) scale (Spitzer et al. 2006), which assesses frequency of generalized anxiety disorder symptoms in the past 2 weeks. Each item of both the CESD‐R‐10 and GAD‐7 is scored from 0–3 and summed, with possible final scores ranging from 0–30 or 0–21, respectively, and higher scores representing greater severity of symptoms. The internal consistency of the CESD‐R‐10 and GAD‐7 measures of this study is high with Cronbach's α = 0.88 and α = 0.92, respectively.

Alcohol use was measured using the Alcohol Use Disorders Identification Test‐Consumption (AUDIT‐C) (Bush et al. 1998), a 3‐item tool which screens for hazardous drinking or alcohol use disorder. Possible scores range from 0–12, with higher scores indicating more hazardous alcohol use.

We identified drug use using the 2‐part Tobacco, Alcohol, Prescription medication, and other Substance use (TAPS) tool (McNeely et al. 2016). TAPS‐1 identifies use in the past 12 months, and screen‐positives are directed to complete TAPS‐2, which assesses risk level based on the past 3 months. Each substance is scored on a scale of 0–3 where 0 is defined as no use in the past 3 months (we also coded TAPS‐1 screen‐negatives as 0). For marijuana, we defined a score of ≥ 1 as moderate‐risk and a score of ≥ 2 as higher‐risk. For other substances (stimulants, opioids, and sedatives), we defined a score of ≥ 1 as higher risk.

### 2.4 Statistical Analysis

Descriptive statistics characterized participants by housing status. Bivariate associations were tested using chi‐square tests and independent‐samples *t*‐tests. For outcomes demonstrating a bivariate association with housing status with a *p*‐value < 0.20 (Hosmer et al. 2013), we built multivariable regression models to assess the relationship between housing status and IPV, health outcomes, and risk behaviours, controlling for covariates including ADI, region, race/ethnicity, age, and education. More specifically, we used linear regression for continuous outcomes (IPV, depression, and anxiety), multinomial logistic regression for STIs/HIV (positive/reactive, negative/non‐reactive, and missing/incomplete) and marijuana (no use, moderate‐risk use, and higher‐risk use), and binary logistic regression for other drug use (no use, higher‐risk use). To account for the hierarchical structure of the data, we fitted mixed‐effects models, where 1626 participants (Level 1) were nested within 51 states (Level 2). In regression models, we used the national ADI ranking in quartiles, with quartile 1 representing the least deprived neighbourhoods (ADI 1–25) and quartile 4 representing the most deprived (ADI 76–100). Regression coefficients, relative risk ratios (RRRs), odds ratios (ORs), and associated 95% confidence intervals were reported to assess the strength and direction of the multivariable associations for linear regression, multinomial logistic regression, and binary logistic regression, respectively. Before building final multivariable regression models, we assessed the multicollinearity of all independent variables (housing instability and all potential covariates including age, race and ethnicity, education, employment status, region, income, and ADI); we removed employment status from the final models due to the presence of high multicollinearity (variance inflation factor [VIF] > 3). Because individual and neighbourhood‐level socioeconomic indicators inherently share significant variance, we applied a conservative VIF cutoff of 3.0 to protect against over‐controlling and multicollinearity bias, preserving the parsimony and stability of the primary explanatory variable, housing instability (Zuur et al. 2010). Appendix A; Table A1 presents VIF values of all independent variables (< 3) from the final regression models.

We examined patterns of missing data and found no discernible patterns. Given that missing data for variables included in the regression models were minimal (< 5% for each variable), we applied listwise deletion in the final mixed‐effects regression models. For IPV analyses, missing item counts were imputed proportionally when ≥ 70% of items were non‐missing. For participants with at least one missing item, the total count of positive items was imputed by multiplying the observed sum by a factor of 15 divided by the number of non‐missing items. Data analysis was conducted in SAS 9.4 (SAS 2013).

## 3 Results

A total of 1626 women with complete housing data between July 2023 and March 2025 were included. Women were from all 50 states, Washington, D.C., and 3 U.S. territories. Sixteen percent (*n* = 261) reported current housing instability, including 3.1% (*n* = 51) who were presently unhoused. Housing instability was associated with significant socioeconomic disadvantage, including lower income, higher unemployment, and lower educational attainment. Table 1 presents the full demographic characteristics.

**TABLE 1:** Demographic and socioeconomic characteristics of participants.

		Housing unstable *n* (%)	Housing stable *n* (%)	Overall *n* (%)	*p*
		*n* = 261	*n* = 1365	*n* = 1626	
Race^a^	White	97 (37.16)	551 (40.37)	648 (39.85)	0.0011
	Non‐hispanic white	73 (27.97)	373 (27.33)	446 (27.43)	
	Hispanic white	24 (9.20)	178 (13.04)	202 (12.42)	
	Black	85 (32.57)	433 (31.72)	518 (31.86)	
	Non‐hispanic black	72 (27.59)	403 (29.52)	475 (29.21)	
	Hispanic black	13 (4.98)	30 (2.20)	43 (2.64)	
	Hispanic/Latina (any race)	56 (21.46)	291 (21.32)	347 (21.34)	
	Multiracial	45 (17.24)	193 (14.14)	238 (14.64)	
	Native American	10 (3.83)	27 (1.98)	37 (2.28)	
	Non‐hispanic native American	4 (1.53)	12 (0.88)	16 (0.98)	
	Hispanic native American	6 (2.30)	15 (1.10)	21 (1.29)	
	Asian	3 (1.15)	81 (5.93)	84 (5.17)	
	Non‐hispanic Asian	3 (1.15)	78 (5.71)	81 (4.98)	
	Hispanic Asian	0 (0.00)	3 (0.22)	3 (0.18)	
	Other (non‐hispanic)	3 (1.15)	8 (0.59)	11 (0.68)	
	Native hawaiian/pacific Islander (non‐hispanic)	2 (0.77)	1 (0.07)	3 (0.18)	
	Unknown	2 (0.77)	3 (0.22)	5 (0.31)	
Age (years)	14–17	0 (0.00)	6 (0.44)	6 (0.37)	0.0011
	18–29	56 (21.46)	471 (34.51)	527 (32.41)	
	30–39	119 (45.59)	528 (38.68)	647 (39.79)	
	40–49	75 (28.74)	291 (21.32)	366 (22.51)	
	50–59	11 (4.21)	61 (4.47)	72 (4.43)	
	60–69	0 (0.00)	3 (0.22)	3 (0.18)	
	Unknown	0 (0.00)	5 (0.37)	5 (0.31)	
Education	Less than high school	8 (3.07)	22 (1.61)	30 (1.85)	0.0077
	High school diploma or GED	58 (22.22)	207 (15.16)	256 (15.74)	
	More than high school	194 (74.33)	1134 (83.08)	1328 (81.67)	
	Unknown	1 (0.38)	2 (0.15)	3 (0.18)	
Employment status	Unemployed (seeking work)	66 (25.29)	165 (12.09)	231 (14.21)	< 0.0001
	Unemployed (not seeking work)	45 (17.24)	198 (14.51)	243 (14.94)	
	Employed full‐time	63 (24.14)	696 (50.99)	759 (46.68)	
	Part‐time or temporary work	87 (33.33)	295 (21.61)	382 (23.49)	
	Unknown	0 (0.00)	11 (0.81)	11 (0.68)	
Annual household income (USD), median (IQR)		25,000 (14,000‐40,000)	40,000 (19,000‐65,000)	38,000 (17,500−62,500)	< 0.0001
Geographic region^b^	South	102 (39.08)	577 (42.72)	679 (41.76)	0.3442
	West	64 (24.52)	262 (19.19)	326 (20.05)	
	Midwest	45 (17.24)	232 (17.00)	277 (17.04)	
	Northeast	41 (15.71)	230 (16.85)	271 (16.67)	
	U.S. Territories	9 (3.45)	63 (4.62)	72 (4.43)	
	Puerto Rico	9 (3.45)	60 (4.40)	69 (4.24)	
	Guam	0 (0.00)	1 (0.07)	1 (0.06)	
	U.S. Virgin islands	0 (0.00)	2 (0.15)	2 (0.12)	
Area deprivation index, mean (SD)		51.63 (± 28.98)	48.28 (± 28.26)	48.83 (± 28.39)	0.09

^a^ Participants could select multiple options.

^b^ South: AL, AR, DE, DC, FL, GA, KY, LA, MD, MS, NC, OK, SC, TN, TX, VA, WV; West: AK, AZ, CA, CO, HI, ID, MT, NV, NM, OR, UT, WA, WY; Midwest: IL, IN, IA, KS, MI, MN, MO, NE, ND, OH, SD, WI; Northeast: CT, ME, MA, NH, NJ, NY, PA, RI, VT.

In bivariate analyses (Table 2), housing instability was associated with greater IPV exposure, higher depression and anxiety scores, higher STI/HIV positivity, and greater current use of marijuana, stimulants, opioids, and sedatives. There was no significant association with alcohol use.

**TABLE 2:** Bivariate relationship between housing status and health outcomes.

	Housing unstable mean (SD)/*n* (%)	Housing stable mean (SD)/*n* (%)	Overall mean (SD)/*n* (%)	*p*
	*n* = 261	*n* = 1365	*n* = 1626	
IPV	7.09 (± 5.13)	4.27 (± 4.68)	4.71 (± 4.85)	< 0.001
Depression	16.04 (± 5.40)	12.6 (± 5.03)	13.14 (± 5.24)	< 0.001
Anxiety	11.08 (± 5.72)	7.54 (± 5.46)	8.11 (± 5.65)	< 0.001
STI^a^				< 0.001
Any reactive/positive	25 (9.58%)	73 (5.35%)	98 (6.03%)	
HIV	1 (0.38%)	2 (0.15%)	3 (0.18%)	
Syphilis	4 (1.53%)	7 (0.51%)	11 (0.68%)	
Chlamydia	9 (3.45%)	26 (1.90%)	35 (2.15%)	
Gonorrhoea	1 (0.38%)	6 (0.44%)	7 (0.43%)	
Trichomoniasis	11 (4.21%)	34 (2.49%)	45 (2.77%)	
Non‐reactive/negative	203 (77.78%)	1196 (87.62%)	1399 (86.04%)	
Missing/incomplete	33 (12.64%)	96 (7.03%)	129 (7.93%)	
Alcohol	3.25 (2.42)	3.09 (1.94)	3.11 (2.02)	0.35
Marijuana				< 0.001
No use	103 (39.46%)	650 (47.62%)	753 (46.31%)	
Moderate‐risk use	46 (17.62%)	363 (26.59%)	409 (25.15%)	
High‐risk use	112 (42.91%)	352 (25.79%)	464 (28.54%)	
Stimulants				< 0.001
No use	209 (80.08%)	1252 (91.72%)	1461 (89.85%)	
Use	52 (19.92%)	113 (8.27%)	165 (10.15%)	
Opioids				< 0.001
No use	236 (90.42%)	1321 (96.78%)	1557 (95.76%)	
Use	25 (9.58%)	44 (3.22%)	69 (4.24%)	
Sedatives				< 0.001
No use	236 (90.42%)	1303 (95.46%)	1539 (94.64%)	
Use	25 (9.58%)	62 (4.54%)	87 (5.35%)	

^a^ Chi‐square analysis was conducted on overall STI status only. Breakdown by STI type was not analysed due to insufficient sample size.

Multivariable models (Tables 3 and 4) indicated that housing instability remained significantly associated with higher IPV (B = 2.43, 95% CI = 1.68–3.18), depression (B = 3.25, 95% CI = 2.61–3.88), and anxiety (B = 3.38, 95% CI = 2.60–4.15) after adjusting for covariates. Housing instability was also associated with an increased likelihood of testing positive for STI/HIV (RRR = 1.85, 95% CI = 1.16–2.97), current higher‐risk marijuana use (RRR = 2.02, 95% CI = 1.38–2.95), stimulant use (OR = 2.31, 95% CI = 1.45–3.69), and opioid use (OR = 2.72, 95% CI = 1.33–5.56). There was no significant association with moderate‐risk marijuana use.

**TABLE 3:** Multivariable linear regression analysis.

			Estimate (95% CI)	Pr > |*Z*|
IPV	Housing instability		2.43 (1.68, 3.18)	< 0.0001
	Race	White	Ref^a^	
		Black	−1.49 (−2.31, −0.68)	0.0003
		Hispanic	−0.87 (−1.64, −0.10)	0.0260
		Asian	−1.46 (−2.75, −0.18)	0.0253
		Other	−0.30, (−1.08, −0.48)	0.4534
	Age		0.05 (−1.08, 0.48)	0.0006
	Education	< High school	Ref	
		≥ High school	−0.20 (−0.90, 0.50)	0.5828
	Income	≥ $50,000	Ref	
		< $50,000	0.49 (−0.20, 1.19)	0.1649
	Region	South	Ref	
		West	1.09 (0.13, 2.05)	0.0259
		Midwest	−0.30 (−1.19, 0.59)	0.5108
		Northeast	−0.22 (−1.30, 0.87)	0.6951
		Other	−0.31 (−2.15, 1.54)	0.7435
	ADI	< 25%	Ref	
		25%–50%	0.49 (−0.32, 1.30)	0.2378
		50%–75%	0.97 (0.04, 1.89)	0.0406
		> 75%	1.50 (0.47, 2.53)	0.0045
Depression	Housing instability		3.25 (2.61, 3.88)	< 0.0001
	Race	White	Ref	
		Black	−0.87 (−1.46, −0.28)	0.0039
		Hispanic	−0.74 (−1.23, −0.25)	0.0031
		Asian	0.48 (−1.91, −0.03)	0.0430
		Other	0.20 (−0.61, 1.02)	0.6240
	Age		−0.05 (−0.07, −0.03)	< 0.0001
	Education	< High school	Ref	
		≥ High school	−0.36 (−0.96, 0.24)	0.2397
	Income	≥ $50,000	Ref	
		< $50,000	0.70 (0.21, 1.19)	0.0049
	Region	South	Ref	
		West	0.72 (−0.06, 1.50)	0.0722
		Midwest	0.23 (−0.53, 1.00)	0.5503
		Northeast	0.43 (−0.01, 0.87)	0.0567
		Other	0.33 (−1.40, 0.74)	0.5405
	ADI	< 25%	Ref	
		25%–50%	0.14 (−0.54, 0.82)	0.6916
		50%–75%	0.05 (−0.55, 0.66)	0.8673
		> 75%	−0.17 (−1.02, 0.68)	0.6988
Anxiety	Housing instability		3.38 (2.60, 4.15)	< 0.0001
	Race	White	Ref	
		Black	−1.67 (−2.34, −1.01)	< 0.0001
		Hispanic	−0.75 (−1.49, −0.02)	0.0450
		Asian	−1.16 (−2.17, −0.14)	0.0251
		Other	−0.22 (−1.03, 0.59)	0.5959
	Age		−0.05 (−0.08, −0.02)	0.0003
	Education	< High school	Ref	
		≥ High school	−0.61 (−1.14, −0.08)	0.0242
	Income	≥ $50,000	Ref	
		< $50,000	0.74 (0.07, 1.40)	0.0293
	Region	South	Ref	
		West	0.54 (−0.28, 1.35)	0.1965
		Midwest	0.16 (−0.75, 1.06)	0.7346
		Northeast	0.23 (−0.60, 1.06)	0.5947
		Other	1.21 (−0.24, 2.67)	0.1031
	ADI	< 25%	Ref	
		25%–50%	0.56 (−0.15, 1.26)	0.1208
		50%–75%	0.57 (−0.17, 1.32)	0.1300
		> 75%	0.81 (−0.15, 1.76)	0.0968

^a^ “Ref” indicates reference category.

**TABLE 4:** Multivariable logistic regression analysis.

			Odds ratio (95% CI)	Pr > |*t*|
Positive/reactive STI^a^	Housing instability		1.85 (1.16, 2.97)	0.0110
	Race	White	Ref	
		Black	1.55 (0.87, 2.74)	0.1321
		Hispanic	1.72 (0.98, 3.02)	0.0596
		Asian	0.75 (0.19, 2.93)	0.6757
		Other	1.76 (0.84, 3.64)	0.1264
	Age		1.00 (0.97, 1.02)	0.7275
	Education	< High school	Ref	
		≥ High school	0.69 (0.41, 1.16)	0.1542
	Income	≥ $50,000	Ref	
		< $50,000	1.21 (0.74, 2.00)	0.4328
	Region	South	Ref	
		West	0.76 (0.46, 1.26)	0.2835
		Midwest	0.86 (0.54, 1.37)	0.5160
		Northeast	0.54 (0.33, 0.90)	0.0174
		Other	1.26 (0.21, 7.44)	0.7951
	ADI	< 25%	Ref	
		25%–50%	1.14 (0.63, 2.05)	0.6605
		50%–75%	0.97 (0.47, 1.98)	0.9209
		> 75%	1.67 (0.83, 3.35)	0.1485
Moderate‐risk marijuana use	Housing instability		0.87 (0.57, 1.31)	0.4842
	Race	White	Ref	
		Black	0.94 (0.62, 1.43)	0.7643
		Hispanic	0.79 (0.54, 1.16)	0.2171
		Asian	1.50 (0.96, 2.33)	0.0738
		Other	1.43 (0.97, 2.10)	0.0719
	Age		0.98 (0.96, 0.99)	0.0032
	Education	< High school	Ref	
		≥ High school	1.32 (0.94, 1.86)	0.1110
	Income	≥ $50,000	Ref	
		< $50,000	1.12 (0.87, 1.44)	0.3845
	Region	South	Ref	
		West	1.49 (0.90, 2.47)	0.1180
		Midwest	1.66 (1.26, 2.19)	0.0006
		Northeast	1.30 (1.04, 1.62)	0.0199
		Other	1.41 (0.70, 2.87)	0.3323
	ADI	< 25%	Ref	
		25%–50%	0.81 (0.59, 1.12)	0.2034
		50%–75%	0.57 (0.39, 0.83)	0.0041
		> 75%	0.76 (0.54, 1.08)	0.1184
Higher‐risk marijuana use	Housing instability		2.02 (1.38, 2.95)	0.0005
	Race	White	Ref	
		Black	1.25 (0.85, 1.85)	0.2480
		Hispanic	0.96 (0.66, 1.41)	0.8310
		Asian	1.09 (0.52, 2.29)	0.8110
		Other	1.40 (0.93, 2.11)	0.1044
	Age		0.97 (0.95, 0.99)	0.0012
	Education	< High school	Ref	
		≥ High school	0.87 (0.62, 1.22)	0.4116
	Income	≥ $50,000	Ref	
		< $50,000	1.17 (0.86, 1.59)	0.3156
	Region	South	Ref	
		West	1.38 (0.91, 2.10)	0.1278
		Midwest	1.53 (1.17, 2.01)	0.0027
		Northeast	1.51 (0.97, 2.35)	0.0684
		Other	1.41 (0.61, 3.29)	0.4154
	ADI	< 25%	Ref	
		25%–50%	0.95 (0.66, 1.37)	0.7754
		50%–75%	1.09 (0.79, 1.49)	0.6060
		> 75%	1.09 (0.76, 1.57)	0.6439
Stimulant use (Cocaine, crack, methamphetamine)	Housing instability		2.31 (1.45, 3.69)	0.0007
	Race	White	Ref	
		Black	0.40 (0.24, 0.68)	0.0009
		Hispanic	0.57 (0.38, 0.87)	0.0092
		Asian	1.20 (0.62, 2.32)	0.5763
		Other	1.06 (0.60, 1.87)	0.8447
	Age		1.04 (1.01, 1.07)	0.0063
	Education	< High school	Ref	
		≥ High school	0.58 (0.37, 0.91)	0.0181
	Income	≥ $50,000	Ref	
		< $50,000	1.05 (0.73, 1.51)	0.7833
	Region	South	Ref	
		West	2.33 (1.49, 3.63)	0.0004
		Midwest	0.77 (0.51, 1.14)	0.1859
		Northeast	0.91 (0.50, 1.68)	0.7687
		Other	1.13 (0.15, 8.74)	0.9086
	ADI	< 25%	Ref	
		25%–50%	0.65 (0.42, 1.02)	0.0610
		50%–75%	1.29 (0.93, 1.80)	0.1270
		> 75%	1.05 (0.62, 1.79)	0.8489
Opioid use (Heroin, prescription opioids)	Housing instability		2.72 (1.33, 5.56)	0.0069
	Race^b^	White	Ref	
		Black	1.39 (0.71, 2.71)	0.3283
		Hispanic	2.23 (1.08, 4.59)	0.0308
		Other	1.52 (0.68, 3.40)	0.3034
	Age		1.04 (1.00, 1.07)	0.0353
	Education	< High school	Ref	
		≥ High school	1.05 (0.54, 2.05)	0.8784
	Income	≥ $50,000	Ref	
		< $50,000	1.37 (0.76, 2.46)	0.2946
	Region	South	Ref	
		West	1.11 (0.64, 1.92)	0.7164
		Midwest	0.48 (0.29, 0.80)	0.0054
		Northeast	0.76 (0.30, 1.88)	0.5382
		Other	1.51 (0.37, 6.19)	0.5591
	ADI	< 25%	Ref	
		25%–50%	1.46 (0.64, 3.29)	0.3597
		50%–75%	2.76 (1.36, 5.60)	0.0060
		> 75%	2.47 (0.99, 6.14)	0.0525

^a^ We report positive/reactive STI compared to negative/non‐reactive STI in this table; however, our model also included missing/incomplete results.

^b^ Asian was combined with Other due to insufficient sample size for independent reporting.

## 4 Discussion

In this large, geographically diverse cohort, housing instability was strongly associated with greater IPV exposure, worsened mental health, an increased likelihood of STI/HIV diagnosis, and higher‐risk drug use. Although our study design did not allow us to determine causality, our results align with prior literature suggesting that the relationship between housing instability and IPV is bidirectional. Abusive partners may limit women's financial independence and constrain their ability to maintain stable housing (Daoud et al. 2016). Conversely, housing instability increases vulnerability to revictimization and sexual risk‐taking (Wenzel et al. 2004). The 85% higher relative risk of STI/HIV positivity among unstably housed women indicates the importance of integrating housing supports into STI/HIV prevention strategies (Grome et al. 2021).

There is well‐established research linking housing instability with poor mental health outcomes, including depression, anxiety, and substance use disorder in high‐income countries (Baker et al. 2020). Controlling for sociodemographic characteristics, we found that women experiencing housing instability had greater depression and anxiety symptomatology and were more than twice as likely to report current higher‐risk use of marijuana, stimulants, or opioids compared with women with stable housing. Stigma and lack of access to mental health care have been shown to further contribute to depressive symptoms among unstably housed individuals (Dobbins et al. 2024). Substance use may arise as a coping mechanism during periods of housing instability, in turn exacerbating mental health symptoms, increasing the risk of overdose and violence victimization, depleting social and economic capital, and further reducing access to stable housing (O'Shaughnessy et al. 2024). Furthermore, prior research has identified an elevated risk of HIV and other infectious disease acquisition among unhoused people who use drugs compared with those with stable housing (Marcus et al. 2020).

Previous studies have shown that prior homelessness was associated with an increased likelihood of alcohol use disorder and severe alcohol symptoms (Moss et al. 2020). Importantly, hazardous alcohol use is linked to behavioural and biological processes that exacerbate risk‐taking behaviours, such as unprotected sex with multiple partners, and that lead to poor health outcomes including increased risk for HIV and STI acquisition (Hahn and Samet 2010; Scott‐Sheldon et al. 2016). However, we found no association between housing status and alcohol consumption. Alcohol use was included as a risk factor for HIV in our inclusion criteria, and our sample could demonstrate greater alcohol use across both groups compared to the general population.

Overall, our findings emphasize the importance of integrated support for women experiencing housing instability. Housing‐focused interventions should extend beyond referrals for temporary shelter and include coordinated, trauma‐informed, and gender‐responsive models of care. Such approaches may include routine screening for housing instability in emergency departments, community health centres, sexual health clinics, behavioural health programmes, substance use treatment settings, and shelter‐based services; warm handoffs to housing navigation and legal advocacy; integration of IPV safety planning; linkage to mental health and substance use treatment; harm reduction services; and access to HIV/STI testing, prevention, and treatment. Nurses and advanced practice providers are well positioned to identify women experiencing housing instability, assess co‐occurring health and safety needs, and connect women to multidisciplinary services.

### 4.1 Limitations

Our social media recruitment strategy may have introduced selection bias. Additionally, because the study required mailing materials and Zoom‐based data collection, women with the most extreme housing precarity or IPV‐related safety concerns may have been underrepresented. Social desirability and recall bias may also have affected self‐reported measures of IPV, substance use, or mental health. Future research should evaluate whether integrated housing, IPV, behavioural health, substance use, and sexual health interventions improve outcomes for women experiencing housing instability. Longitudinal studies are also needed to clarify temporal pathways and to determine whether different levels of housing precarity, including being unhoused versus being worried about losing housing, are associated with different health risks and intervention needs. Additional relevant factors, including food insecurity, physical health outcomes, LGBTQ+ identity, and healthcare access, warrant further research.

## 5 Conclusion

In this geographically diverse sample of women across the U.S. and its territories, housing instability was associated with greater IPV exposure, higher depression and anxiety symptoms, an increased likelihood of STI/HIV diagnosis, and higher‐risk marijuana, stimulant, and opioid use. These findings suggest that housing instability should be treated not only as a social risk factor but also as an important marker for intersecting violence, behavioural health, substance use, and sexual health needs among women.

## Author Contributions

Conceptualization and funding acquisition: Rebecca Schnall, Amy K. Johnson, and Mirjam‐Colette Kempf; formal analysis: Claudia L. Michaels, Jianfang Liu, and Jean Jimenez; investigation: Claudia L. Michaels and Maeve Brin; methodology: Rebecca Schnall, Amy K. Johnson, and Mirjam‐Colette Kempf; resources: Rebecca Schnall, Amy K. Johnson, Mirjam‐Colette Kempf, and Jianfang Liu; supervision: Rebecca Schnall, Amy K. Johnson, and Mirjam‐Colette Kempf; writing – original draft: Claudia L. Michaels and Jianfang Liu; writing – review and editing: Claudia L. Michaels, Jianfang Liu, Jean Jimenez, Maeve Brin, Nancy Glass, Amy K. Johnson, Mirjam‐Colette Kempf, and Rebecca Schnall. All authors read and approved the final manuscript. Generative AI tools were not used at any stage of writing or analysis.

## Funding

Research reported in this publication was supported by the National Institute of Allergy and Infectious Diseases of the National Institute of Health under award number R01AI172469. The content is solely the responsibility of the authors and does not necessarily represent the official views of the NIH.

## Ethics Statement

The study was conducted in accordance with the Declaration of Helsinki, and approved by the Columbia University Institutional Review Board (IRB) under protocol AAAU2650 on August 4, 2022. Study participants provided written informed consent prior to enrollment. Researchers followed institutional policies on data collection and management procedures.

## Consent

The authors have nothing to report.

## Conflicts of Interest

The authors declare no conflicts of interest.

## Data Availability

The data that support the findings of this study are available from the corresponding author upon reasonable request.

## Appendix A

**TABLE A1:** Variance inflation factor assessing multicollinearity of independent variables in final regression models.

Variable		Variance inflation^a^
Housing instability		1.032
Race	Black	1.540
	Hispanic/Latina	1.512
	Other	1.511
Age		1.094
Education	≥ High school	1.042
Income	< $50,000	1.025
Region	West	1.330
	Midwest	1.185
	Northeast	1.237
ADI	25%–50%	1.435
	50%–75%	1.586
	> 75%	1.721

^a^ Dummy variables of categorical variables not including the reference groups were created to generate VIF values.
